# Supplementary material for: A Bioinformatics Investigation of Hub Genes Involved in Treg Migration and Its Synergistic Effects, Using Immune Checkpoint Inhibitors for Immunotherapies
Source: Int J Mol Sci. 2024 Aug 28;25(17):9341. doi: 10.3390/ijms25179341 (PMC11395080; doi:10.3390/ijms25179341)
Supplement: Supplementary file 1 [file ijms-25-09341-s001.zip › ijms-3171204-supplementary.pdf]

Supplementary Materials

Table S1. TCGA Abbreviations

Abbreviation	Cancer Type
LAML	Acute Myeloid Leukemia
ACC	Adrenocortical carcinoma
BLCA	Bladder Urothelial Carcinoma
LGG	Brain Lower Grade Glioma
BRCA	Breast invasive carcinoma
CESC	Cervical squamous cell carcinoma and endocervical adenocarcinoma
CHOL	Cholangiocarcinoma
LCML	Chronic Myelogenous Leukemia
COAD	Colon adenocarcinoma
ESCA	Esophageal carcinoma
GBM	Glioblastoma multiforme
HNSC	Head and Neck squamous cell carcinoma
KICH	Kidney Chromophobe
KIRC	Kidney renal clear cell carcinoma
KIRP	Kidney renal papillary cell carcinoma
LIHC	Liver hepatocellular carcinoma
LUAD	Lung adenocarcinoma
LUSC	Lung squamous cell carcinoma
DLBC	Lymphoid Neoplasm Diffuse Large B-cell Lymphoma
MESO	Mesothelioma
MISC	Miscellaneous
OV	Ovarian serous cystadenocarcinoma
PAAD	Pancreatic adenocarcinoma
PCPG	Pheochromocytoma and Paraganglioma
READ	Rectum adenocarcinoma
PRAD	Prostate adenocarcinoma
SARC	Sarcoma
SKCM	Skin Cutaneous Melanoma
STAD	Stomach adenocarcinoma
TGCT	Testicular Germ Cell Tumors
THYM	Thymoma
THCA	Thyroid carcinoma
UCS	Uterine Carcinosarcoma
UCEC	Uterine Corpus Endometrial Carcinoma
UVM	Uveal Melanoma

Table S2. Overlapping Differentially Expressed Genes Across Three GEO Datasets

gene name	baseMean	log2FoldChange	lfcSE	stat	pvalue	padj
CD177	531.8484	6.173373	0.583646	10.57725	3.80E-26	4.48E-22
SH3RF2	206.9715	4.687677	0.70038	6.693044	2.19E-11	4.95E-09
ACTG2	785.1331	3.867379	0.710671	5.441869	5.27E-08	5.70E-06
TMPRSS6	232.0943	3.801275	0.793644	4.789646	1.67E-06	0.000114
IL1R2	7309.189	3.467286	0.48567	7.139177	9.39E-13	2.91E-10
LAPTM4B	2759.652	3.132174	0.401037	7.81019	5.71E-15	3.20E-12
GCNT1	1595.537	2.961397	0.393689	7.522166	5.39E-14	2.30E-11
TNFRSF9	17120.11	2.917921	0.332335	8.780068	1.63E-18	2.75E-15
SOX4	1208.728	2.683295	0.317007	8.464455	2.57E-17	2.76E-14
TNFRSF8	1186.587	2.411056	0.453714	5.314048	1.07E-07	1.10E-05
CCR8	9545.405	2.354741	0.519271	4.534703	5.77E-06	0.000312
CD80	886.2005	2.28728	0.563428	4.059575	4.92E-05	0.00188
EBI3	2285.78	2.07913	0.318219	6.53365	6.42E-11	1.35E-08
TNFRSF4	19650.24	2.060931	0.443874	4.643053	3.43E-06	0.000207
SYNGR2	22232.71	2.016169	0.257746	7.822302	5.19E-15	3.05E-12
CEP55	1829.11	1.966147	0.299996	6.553915	5.60E-11	1.20E-08
IL2RA	22295	1.908044	0.245531	7.771088	7.78E-15	3.99E-12
LY75	1265.479	1.833217	0.497785	3.68275	0.000231	0.006177
TNFRSF18	25275.06	1.634569	0.423462	3.860014	0.000113	0.003619
ADPRH	692.6585	1.560968	0.337144	4.62997	3.66E-06	0.000218
FLNB	715.5796	1.496626	0.377351	3.966138	7.30E-05	0.002607
CTNNA1	3056.457	1.426296	0.445649	3.200495	0.001372	0.024083
TYMP	11683.4	1.395248	0.434455	3.211492	0.00132	0.023389
CRADD	1024.934	1.360158	0.311127	4.371719	1.23E-05	0.000587
BATF	16789.5	1.325111	0.255905	5.178141	2.24E-07	2.02E-05
IL12RB2	8003.881	1.310859	0.327444	4.003311	6.25E-05	0.002319
HIP1	228.7819	1.299542	0.317682	4.090694	4.30E-05	0.0017
ACOT9	2710.978	1.292832	0.317625	4.070305	4.70E-05	0.001837
HTATIP2	6481.5	1.176351	0.389534	3.019892	0.002529	0.038682
TRAF3	5218.621	1.172996	0.208248	5.632677	1.77E-08	2.13E-06
TRAF1	4097.939	1.160004	0.382253	3.034648	0.002408	0.037144
SEC14L1	3281.439	1.015556	0.211142	4.809833	1.51E-06	0.000105
GOT2	4690.262	0.853712	0.217369	3.92747	8.58E-05	0.002914
RFK	625.2683	0.760796	0.253817	2.997426	0.002723	0.040959
IKBKE	3952.487	0.607854	0.195937	3.102299	0.00192	0.031023
KLF12	726.1599	-0.89755	0.227171	-3.95097	7.78E-05	0.002736
MAP3K3	1149.03	-1.22009	0.363975	-3.35212	0.000802	0.016038

IQGAP2	3814.012	-1.27796	0.290375	-4.40106	1.08E-05	0.000531
RCBTB2	446.0752	-1.41188	0.36973	-3.81867	0.000134	0.004116
SMAD3	371.288	-1.45538	0.442552	-3.28862	0.001007	0.019128
SYNE1	2516.708	-1.51059	0.328533	-4.59799	4.27E-06	0.000248
PRKCA	70.10504	-1.60013	0.544895	-2.93658	0.003319	0.046943
SERINC5	334.5444	-1.69765	0.278986	-6.08507	1.16E-09	1.85E-07
GIMAP7	2805.693	-1.83132	0.362849	-5.04706	4.49E-07	3.77E-05
LPAR6	1045.511	-2.09354	0.545379	-3.83869	0.000124	0.003852
CCR7	8106.123	-2.14372	0.566296	-3.78552	0.000153	0.004645
CAMK1D	282.4837	-2.23349	0.30179	-7.40081	1.35E-13	5.14E-11
TEC	58.95809	-2.38009	0.61921	-3.84375	0.000121	0.003796
TXK	1093.184	-2.50294	0.701426	-3.56836	0.000359	0.008689
TC2N	2299.726	-2.87951	0.666767	-4.31862	1.57E-05	0.00072
GSAP	194.1944	-2.93191	0.383828	-7.63861	2.20E-14	1.03E-11
TCF7	481.1734	-3.15534	0.682237	-4.62499	3.75E-06	0.000222
PLAC8	47.64539	-3.24741	0.998655	-3.25179	0.001147	0.021206
LRMP	1231.484	-3.51052	0.408947	-8.58429	9.14E-18	1.20E-14
MYO7A	2367.285	-3.56048	0.785946	-4.53018	5.89E-06	0.000317
IRF8	152.2222	-3.5626	0.829247	-4.29619	1.74E-05	0.000781
ATP10A	151.4424	-4.84435	1.011107	-4.79113	1.66E-06	0.000114

baseMean, The average expression level of the gene across all samples in the dataset; log2FoldChage, The logarithm (base 2) of the fold change in expression of the gene between two conditions; lfcSE, The standard error of the log2 fold change; stat, The test statistic for differential expression, typically derived from a statistical test comparing expression levels between conditions; pvalue, The probability value indicating the likelihood of observing the data; padj, The adjusted p-value, corrected for multiple testing using methods such as the Benjamini-Hochberg procedure;
